# Surgical team dynamics in a reflective team meeting to improve quality of care: qualitative analysis of a shared mental model

**DOI:** 10.1093/bjs/znad111

**Published:** 2023-05-16

**Authors:** Merel J Verhagen, Marit S de Vos, Jan van Schaik, Joost R van der Vorst, Abbey Schepers, Perla J Marang-van de Mheen, Jaap F Hamming

**Affiliations:** Department of Vascular Surgery, Leiden University Medical Centre, Leiden, the Netherlands; Directorate of Quality and Patient Safety, Leiden University Medical Centre, Leiden, the Netherlands; Department of Vascular Surgery, Leiden University Medical Centre, Leiden, the Netherlands; Department of Vascular Surgery, Leiden University Medical Centre, Leiden, the Netherlands; Department of Vascular Surgery, Leiden University Medical Centre, Leiden, the Netherlands; Department of Biomedical Data Sciences, Leiden University Medical Centre, Leiden, the Netherlands; Department of Vascular Surgery, Leiden University Medical Centre, Leiden, the Netherlands

## Introduction

Morbidity and mortality (M&M) conferences are an acclaimed method for achieving case-based learning and improving surgical care^1^. Their educational value is acknowledged, but whether these conferences contribute to systemic improvement is unclear^2^. M&M formats vary widely^3,4^, and are mostly focused on severe adverse events and individual performance, thus lacking consideration of system-level issues or similar cases where successful outcomes were achieved^3,5,6^. To overcome these shortcomings, an adapted weekly M&M meeting was developed at the authors unit^7^. In the adapted meeting, the surgical team collectively reflects both on all recently discharged but also on planned procedures, which is consistent with existing frameworks^8^. Discussing all cases also directs attention to successful outcomes, rather than only the complicated ones. This allows the team to understand how to ensure safety for their patients continuously^9,10^.

The aim of this qualitative study was to investigate how the novel weekly reflective team meeting affects the dynamics of a surgical team and improves the quality of care.

## Methods

Traditionally, the entire Department of Surgery of Leiden University Medical Centre held departmental monthly M&M conferences^11,12^. In 2016, the vascular surgical services implemented a weekly reflective team meeting, which allowed high-frequency discussion of a wider spectrum of patients, rather than only preselected and complicated cases^7^. Most patients admitted to the hospital are thus discussed twice (before and after operation) to ensure both anticipation (for example co-morbidities or specific perioperative points of attention) and evaluation, as inspired by recent developments in safety science^9,10^. Apart from clinical aspects, attention is also paid to the corresponding administrative requirements and logistical issues. A detailed description can be found in the *supplementary material*.

A qualitative study was undertaken between May and June 2021, using semistructured interviews. The medical ethics committee waived the need for medical ethical approval under Dutch law (G20.056). From 45 vascular surgeons, residents, and nurses involved in vascular surgical care, study participants were selected if present at the weekly meeting at least twice a month. Purposive sampling resulted in three vascular surgeons, four residents, two physician assistants, and the head nurse of the ward being interviewed (total 10). The interviews were conducted by two interviewers, who were not involved in clinical care during the study.

Inductive thematic coding was used to identify themes. Thematic saturation, defined by absence of additional themes appearing from the data, was achieved after eight interviews. The theory of shared mental models (SMMs) was used to better understand the mechanism of effect. An SMM can be defined as ‘the organized understanding of relevant knowledge shared by team members’. Healthcare teams with an SMM can work effectively as a team, through enhanced coordination of actions in high-risk and complex circumstances^13–15^. An SMM can be used to assess the likely effectiveness of a team, or as an explanatory model to understand how it is effective^14,16^, as done in the present study.

## Results

**Fig. 1:**
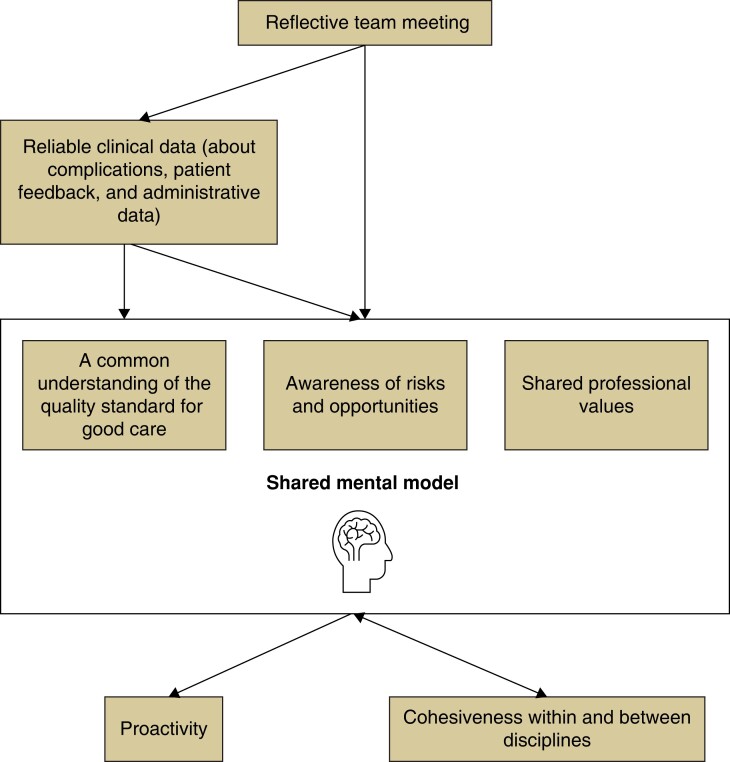
How a reflective team meeting contributes to a shared mental model

The overarching finding was that the reflective team meeting worked to promote an SMM among team members, thereby improving quality of care. Specifically, the meeting facilitated the availability of ‘reliable clinical data’, thereby contributing to ‘a common understanding of the quality standard for good care’, ‘awareness of risks and opportunities’, and shaping ‘shared professional values’ (*Fig. 1*). Together these items formed the team SMM and contributed to the sense of ‘being on the same page’. The SMM in turn enhanced proactivity and cohesiveness among professionals, thereby affecting the ability to deliver better patient care.

Interviewees expressed that the meeting increased data accuracy and completeness (for example complications or patient feedback), as every discharged patient was discussed and missing information added (*Table 1*, quote 1.1). Having more reliable data informed and supported an SMM, as it added to a sense of ‘knowing what you are doing’ and ‘knowing what to look out for’, but also contributed to a shared understanding of what good-quality care entails. Discussing both successful and unsuccessful outcomes was felt to raise awareness about the spectrum of possible outcomes. Participants felt that the meeting contributed to developing a ‘gut feeling’. This could be explained as a shared perception of likely clinical scenarios, and potential risks and contingencies accompanying surgical procedures, thus providing for another part of the SMM (*Table 1*, quote 3.3). The participants’ professional identity was shaped and enforced in the meeting by sharing and assessing values regarding clinical, emotional, and process-related aspects of delivered care. Therefore, this almost served as an audit of clinical cases (*Table 1*, quote 4.1). The opportunity for doctors and nurses to question each other about patients or about experiences with specific clinical challenges was considered supportive for mutual trust and collaboration, thereby contributing to the SMM. At the same time, this perception of being ‘on the same page’ could in turn intensify collaboration and mutual cohesiveness, providing for a reciprocal relationship between the SMM and team cohesion. Moreover, by frequent discussion of similar cases, patterns were recognized and participants became aware of recurring issues (*Table 1*, quote 6.1). Having an SMM seemed to enable the team to develop and support not only a shared language, but also an action-oriented mindset. A more detailed description of the subthemes identified can be found in the *supplementary material*.

**Table 1:** Subthemes and illustrative quotes derived from qualitative analysis

Subtheme	Illustrative Quotes
**1 RELIABLE CLINICAL DATA**	1.1 ‘[..] It is pragmatic: every patient is discussed, and for every patient a complication form is filled out. This provides for a clear picture of what you are doing’ *Surgeon* 1.2 ‘[This meeting] provides for a very accurate representation of what we do. You know exactly what you did in the past year, and you can check how often specific complications occurred. You can be sure that you have everything recorded [in the electronic health records]; even the smallest things. That is what I really like about this system of weekly discussing all cases. Subsequently, I can propose substantiated alterations with fellow colleagues, anaesthesiologists, the operating room manager or even the board of the hospital’ *Surgeon* 1.3 ‘What I think is very valuable, is that everything is registered better and more accurately than in the past. Our complication registration used to be a bit messy, and you knew that it was incomplete for most patients. That was, for instance, because complications were registered two months after taking place. It lost its value completely. Now, registering complications adequately has made patient records up-to-date. The output is better because we register right after the moment of happening, with the incident still fresh in our memory. Moreover, we discuss all registered complications with the team, so everybody can elaborate on “no, that is not how it went” and, “this is what actually happened”. Discussing complications is much more valuable now’ *Physician assistant*
**2 A COMMON UNDERSTANDING OF THE QUALITY STANDARD FOR GOOD CARE**	2.1 ‘Discussing one or two cases [in an M&M conference] is useful, naturally; one can learn from that, given that they are usually selected because they are considered educational. [..] But when you want a department to increase in overall quality, you must bed in something more structural; something that evaluates everything. That is, “How should we round? How did we arrange the logistics? How can we split clinical tasks? Who should carry the pager? Is there anything that needs to be discussed at the outpatient clinic?” you know, those things, which seem insignificant at first’ *Resident* 2.2 ‘This meeting provides insight into our own clinical practice, but it is also a moment to reflect and to converse with each other. Something that you would probably do to a lesser extent, if not making the time once every week’ *Surgeon* 2.3 ‘This forces you to really dive into a case, and provides for everybody to think about, and collectively contemplate on the case. Otherwise, one is a bit more dependent on who is the primary practitioner on the case. I think this forces you to treat a patient as a team, rather than that you are having the situation where one patient “belongs” to one surgeon, and the next patient “belongs” to another surgeon’ *Resident* 2.4 ‘It [the team meeting] is an assessment of how you do it and why. You could actually consider some sort of peer review’ *Physician assistant* 2.5 ‘It so important to look at the things that we achieved. This also means, for instance, a patient [undergoing a complex vascular procedure] who postoperatively has a significant number of complications, but who is eventually discharged without too significant loss of physical functionality. It is too easy to conclude: “28 complications, that is not good”, so to speak. Yet, what we are looking at is actually a very good outcome of a very high-risk and extensive procedure. The patient survived, has no physical loss of function, can now see his grandson grow up, and is happy with his treatment. I think we have done very well here. [..] This is just as important’ *Surgeon*
**3 AWARENESS OF RISKS AND OPPORTUNITIES**	3.1 ‘I think one—and that might not be done so consciously—learns or develops a level of perception about what kind of complications actually occur in certain categories of patients. This then subconsciously influences one's future actions. Of course, you would want that to be a bit more deliberate, or identifiable, yet it is more a feeling that one develops’ *Physician assistant* 3.2 ‘I do not think discussing the occurrence of a pneumonia in a patient directly involves my clinical actions, rather you just become more thoughtful of the fact that you have to be aware that it can happen’ *Resident* 3.3 ‘If you can name a specific learning point you do that, naturally. But apart from that, it is about developing your gut feeling, and it is very difficult how you are going to capture that’ *Surgeon* 3.4 ‘Recognizing patterns is not necessarily something that you can deduct from clinical data. If you assemble everything, then that is just an overview; that is just data. You subsequently will not recognize or remember when a complication did, or did not occur, or why—and you don’t know which clinical scenario was related to it. But discussing cases every week can make you see things that often come back, to subsequently maybe notice a pattern’ *Surgeon*
**4 SHARED PROFESSIONAL VALUES**	4.1 ‘The meeting is foremostly a moment to pass a judgement as a group. For instance, we contemplate as a group by saying: “so this happened; what do we think of that?”’ *Resident* 4.2 ‘You notice that when you have known a patient for 3 years already, and a certain procedure […] Is discussed afterwards, it is hard to be objective. That is just the way it works. You are emotionally involved, and this can make it difficult to argue whether you made a good or suboptimal choice in that case. Not consciously, but that just happens subconsciously. It is good to hear from somebody else: “I was thinking… maybe you could have done that differently” or “what were your deliberations in that moment?” or “explain to us how you came to that decision”’ *Surgeon* 4.3 ‘It creates an opportunity to address each other, to give feedback on things—that is, also on the things that did not go quite as planned. This opportunity is mutual for all participants. Often, neither the moment when something happens is the ideal moment to debate it, nor are the morning rounds. Yet, in the weekly team meeting you create time and space to talk those situations over with each another’ *Nurse*
**5 COHESIVENESS WITHIN AND BETWEEN DISCIPLINES**	5.1 ‘I think that this [weekly team meeting] keeps your team together. It could provide for a fine, possibly additional role within a hospital: to see each other more often, to talk about things. Just to know better from each other what others are busy with’ *Resident* 5.2 ‘But when the nursing staff is present too, and they can share their opinion, everybody is discussing on the same level. You get to know each other a little better. You subsequently work on small projects together, such as for instance a project that was concerning daily measuring anticoagulation therapy at the ward, which was afterwards discussed in the reflective team meeting as well. This improves the overall intercourse. I guess, in a way it removes a barrier’ *Surgeon* 5.3 ‘The thing is, we used to get together with the entire surgical department, and we noticed that, being a super-specialized team nowadays, we were not able to discuss with other subspecialties on the right level anymore. That is, it is difficult to critically question a fellow surgeon about a Whipple-procedure* that I do not perform being a vascular surgeon; it is questionable how valuable that is’ *Surgeon* 5.4 ‘*W*ith us nurses present at the team meeting, it provides for more interconnectedness between the nursing staff and the doctors, aiding in working with each other on good quality of our care delivered. With that, the mutual consultation has become more accessible. If there are problems at the ward, these are usually talked over during the rounds, yet there are always matters that are not specifically patient bound. Discussing these matters in the reflective team meeting facilitates easy coordination and approachable collaboration’ *Nurse*
**6 PROACTIVITY**	6.1 ‘One of the lessons that we learned in the beginning was, for instance, about the interventions with brachial access. At some point we discussed that if you want to use a certain sheath size, you might have to reconsider whether you want to do that percutaneously, given that we were re-operating all the percutaneous interventions. We did not specifically make an overview of the exact number of reinterventions, that is just something that you come across when you discuss cases more frequently. […] That is when you see it’ *Surgeon* 6.2 ‘Subsequently, you should not be afraid to alter a “lesson learned” again. That is, you have to question the “lessons learned”, by saying “alright, we stated this 7 months ago, but I have done it differently in a few recent cases because I noticed that I did not like it. What do we think of it now?” And then you really explore your findings at a high level’ *Surgeon* 6.3 ‘Discussing [cases] only might make a team a little bit better, because you become more alert to the things that are going on. Yet, initiating meaningful improvement projects might actually lower the incidence of complications. For instance, we had the improvement project on measuring the international normalized ratio (INR) daily: we used to experience problems with anticoagulation therapy to a certain extent. We now see these problems occasionally, yet they are detected earlier. Detection is often without a specific clinical cause, that is, without a bleeding or an occluded artery for instance. I think that is a good example of how we now improve’ *Surgeon* 6.4 ‘Well that [registering low severity complications] is perhaps something that we could consider “a dime a dozen”. For instance, a mild hypokalemia or a urinary tract infection might seem trivial, yet this should in fact not matter, as it is not insignificant to the patient. He is definitely suffering from something like that. I think these are the things where you can meaningfully improve your quality of care; the low complexity, and relatively high volume complications’ *Surgeon*

A pancreatoduodenectomy, which is generally performed by a specialized gastrointestinal surgeon.

## Discussion

This study portrayed how modifying traditional a M&M conference into a weekly reflective team meeting contributed to an SMM, and thereby reinforced proactivity and cohesiveness among team members. The aspects of team functioning, and dynamics identified, seem fundamental to the ability to deliver better patient care as a team. Taken together, the reflective team meeting seemed to help the team to stay on top of details, detecting concerns quickly, and taking action if performance was deteriorating or below the standard of good-quality care. Other effects of regular reflection through a team meeting, in comparison to traditional M&M meetings, are discussed in the *supplementary material*.

Only a few studies have investigated SMMs in surgery, despite the potential of these cognitive constructs for teams^17,18^. Similar to the present findings, a recent clinical study^15^ portrayed how an SMM appropriately described the similar perceptions of teamwork quality within a team, and showed how those with a strong SMM experienced enhanced openness of communication and cohesion. This in turn positively affected teamwork and patient care around hospital discharge. Essentially, sharing a social process with a team can contribute to collective intelligence, and development of plausible initiatives based on the team awareness and organization^19^. In surgery, this collective intelligence and awareness could be deployed to help counteract systemic and structural issues, such as shortage of resources (for example staff, beds or theatre time), and the associated ethical stress for a team.

Strengths of this study include that the interviews were conducted by interviewers not involved in implementing the meeting, to avoid interviewees feeling reluctant to express critical opinions. Limitations include that this study involved a single-centre vascular surgery team. Only a limited number of members of this team could be interviewed, given that only regular attendees of the meeting were deemed eligible to discuss the value of, and learning points from, the meeting. Less frequently participating attendees might have provided additional insights regarding reasons for not attending the meeting.

A novel reflective team meeting in surgery, serving to contemplate collectively on everyday care by weekly discussion of all discharged and scheduled patients, appeared instrumental in creating an SMM among team members. Having a shared understanding of knowledge, skills, interactions, and team member responsibilities has a positive effect on team dynamics, and thereby team functioning and the quality of patient care.

## Supplementary Material

znad111_Supplementary_Data

## Data Availability

Data available on request due to privacy/ethical restrictions.
