# Supplementary material for: Surgical team dynamics in a reflective team meeting to improve quality of care: qualitative analysis of a shared mental model
Source: Br J Surg. 2023 May 16;110(10):1271–5. doi: 10.1093/bjs/znad111 (PMC10480032; doi:10.1093/bjs/znad111)
Supplement: znad111_Supplementary_Data [file znad111_supplementary_data.docx]

**Importance of surgical team dynamics in a reflective team meeting to improve quality of care: qualitative analysis portraying a Shared Mental Model**

Merel J. Verhagen, MD.,^1^ Marit S. de Vos, M.D., PhD,^2^ Jan van Schaik, M.D.,^1^ Joost R. van der Vorst, M.D., PhD,^1^ Abbey Schepers, M.D., PhD,^1^ Perla J. Marang-van de Mheen, PhD,^3^ Jaap F. Hamming, M.D., PhD^1^

1. Department of Vascular Surgery, Leiden University Medical Centre, Leiden, The Netherlands

2. Directorate of Quality and Patient Safety, Leiden University Medical Centre, Leiden, The Netherlands

3. Department of Biomedical Data Sciences, Leiden University Medical Centre, Leiden, The Netherlands

**Corresponding author:**

Jaap F. Hamming

Department of Surgery, LUMC

Address: Albinusdreef 2, 2333 ZA Leiden, The Netherlands

E-mail: J.F.Hamming@lumc.nl

**Supplementary Materials - Index**

| **Supplementary Methods** | |  |
| --- | --- | --- |
| Setting | | *pag. 2* |
| Data collection  Data analysis | | *pag. 2*  *pag. 2* |
| **Supplementary Results** | |  |
| Detailed description of qualitative subthemes identified | | *pag. 4* |
| **Supplementary Appendixes** | |  |
| Appendix S1. The value of the reflective team meeting as an alternative format for M&M. | *pag. 6* | |
| **Supplementary Figures and Tables** | |  |
| Box S1. Real case examples of anticipation and evaluation at a surgical reflective team meeting. | | *pag. 7* |
| File S1. Topic list for semi-structured interviews with participants (surgeons, residents and nurses) of the reflective team meeting. | | *pag. 8* |
| **References** | | *pag. 9* |
|  | |  |

**Supplementary Methods: study setting, details on data collection and analysis.**

**Setting**

This study was presented to the Medical Ethics committee, who waived the need for medical ethical approval under Dutch law (G20.056). The consolidated criteria for reporting qualitative research (COREQ) were used to guide reporting.(1) This study was performed at the Division of Vascular Surgery of the Leiden University Medical Centre (LUMC), which is an academic teaching hospital consisting of 882 beds. The vascular service has an average annual inpatient volume of 510 inpatient cases, with a team of four vascular surgeons, seven residents, two physician assistants and a team of rotating ward nurses. Apart from routine vascular care, this division also serves the role of a tertiary centre for the referral of complicated cases, such as those requiring redo surgery, for instance as a result of complex aortic (endo)vascular protheses infections.

Traditionally, the Department of Surgery held departmental monthly M&M’s.(2, 3) In 2016, the vascular services implemented a weekly reflective team meeting, discussing all discharged cases from the previous week, as well as the cases scheduled for the next week.(4) The new format provides for a high-frequency discussion of a wider spectrum of cases, rather than the pre-selected, complicated cases. Moreover, most inpatient cases are discussed twice (pre- and postoperatively) to ensure both anticipation (e.g., comorbidities or specific perioperative points of attention) and evaluation. Apart from clinical aspects, there is also attention for the corresponding administrative requirements and logistic issues. Departmental M&M currently takes place once every 6 weeks to share instructive cases across different surgical services. A more detailed description of the format of the reflective team meeting can be found elsewhere.(4)

**Data collection**

Between May and June 2021, ten 45-minute semi-structured interviews were conducted with healthcare providers participating in the reflective team meeting on a regular basis. From a total of 45 vascular surgeons, residents and nurses who are to some extent involved in vascular surgery care, study participants were selected based on being present at the weekly meeting at least twice a month. Purposive sampling resulted in three vascular surgeons, four residents (of which one senior resident), two physician assistants and the head nurse of the ward being approached for the interviews. This sample reflected different experience levels and roles within the team and how, from their perspective, they view the team meeting. Interviewees were invited to participate in the study by email, informing them about study objectives and confidentiality of interview data. All team members who were approached agreed to participate. Consent was given at the start of the interview.

The interview guide (supplementary file 1) was developed and refined by the authors (MV, MdV, PM, JH). The topics, as well as three clinical cases serving as vignettes, were chosen based on previous observations of the meeting and previous research into M&M conferences.(4, 5) Interviewees were asked about their overall experiences with the reflective team meeting, their ability to learn and improve patient care through this meeting, and about perceived differences with traditional M&M. The vignettes served to stimulate an in-depth discussion and to help elicit interviewees’ opinions. The interviews were performed by two interviewers (a medical doctor (MV), and a research assistant with a background in psychology), who were not involved in clinical care during the study.

**Data analysis**

Interviews were audio recorded, and transcribed verbatim, after which interviewees were offered the opportunity to review their transcript to ensure accuracy of the data. Inductive thematic coding was used for data analysis. Using ATLAS.ti software, open coding was performed by one of the authors (MV). The coded transcripts were reviewed by another author (MdV), to detect inconsistencies or incorrect codes. The encountered discrepancies were discussed until consensus was reached. A provisional list of themes was made by the two coders, which was followed by an iterative process with the other authors (PM, JH) to develop a coding scheme. This coding scheme was then used to code all interviews. Thematic saturation, defined as no additional themes appearing from the data, was achieved after 8 interviews. During the iterative process, the theory of SMM was used to examine the relationships between themes, and, ultimately, gain a deeper understanding of the mechanism by which the meeting worked to improve the quality of care. An SMM can be defined as “the organized understanding of relevant knowledge shared by team members”, and healthcare teams that have an SMM are considered to be able to work as a team effectively, through enhanced coordination of one’s actions in high risk and complex circumstances.(6-8) An SMM can be used to assess a team’s likely effectiveness, to identify difficulties within teams and facilitate understanding on how to overcome these, or as an explanatory model, as was done in the present study.(7, 9, 10) Finally, representative quotes were selected to show coherence and how these supported the specific theme.

**Supplementary Results: Detailed description of qualitative subthemes identified**

**Reliable clinical data and mandatory documentation**

Interviewees expressed that the meeting increased the accuracy and completeness of data such as complications, patient feedback after discharge, and administrative data (e.g., letters to the general practitioner or reimbursement codes). Clinicians experienced that more complications were detected because every discharged case was discussed in the meeting. They also indicated that complications were registered sooner after the event than would have occurred in previous times, and that the meeting provided for a moment to collectively confirm whether the registered complications were correct (Table 1, quote 1.3). Improved registration and verification made interviewees consider the data to be more reliable and therefore more useful as input for clinical practice or research purposes. Moreover, accurate registration added to a sense of ‘knowing what you’re doing’ and ‘knowing what to look out for’. This way, ensuring more reliable clinical data gained through the meeting, informed and supported an SMM by collectively assessing what encompasses good care, whether this was actually delivered to the case discussed, as well as to raise awareness of the likelihood of certain outcomes.

**A common understanding of the quality standard for good care**

Collective reflection on case- and process-specific aspects served the aim of critically appraising the care that was, and will be delivered, which continuously contributed to a shared understanding of what good quality care entails. This was supported by reflecting on team members’ considerations in decisions or actions in a case, as well as on technical aspects that played a role. During these reflections, caregivers frequently compared expectations about a patient’s course with actual events, such as, for instance, when cases were preoperatively anticipated as complex and at risk for complications, but actually experienced an uncomplicated clinical course. This triggered a discussion about possible reasons for this difference and portrayed how a patient’s clinical course is reviewed considering what can, and may be expected (Table 1, quote 2.5). That team members collectively contemplate on whether good care was delivered, seems to only be possible when there is a common comprehension of what that good care entails. Yet, interviewees cautioned that it should be kept in mind that a surgical team appraising the care they provided themselves, might pertain the risk of ‘marking one’s own exam’. In that respect, it could also be valuable to occasionally have another expert joining the team meeting to critically assess cases, or to ask questions from an outsider’s perspective. Lastly, interviewees mentioned the meeting triggered reflection on organizational aspects as well, such as by discussing how clinical tasks are and should be performed (e.g., who should carry the pager) (Table 1, quote 2.1). This also touched upon the common understanding of team functioning needed to complete a task and thereby the SMM.

**Awareness of risks and opportunities**

Discussing both successful as well as unsuccessful outcomes, was felt to raise awareness about the spectrum of possible outcomes. Participants felt that the meeting contributed to the developing a ‘gut feeling’, which could be explained as a shared perception of likely clinical scenarios, and potential risks and contingencies accompanying surgical procedures, providing for another part of the SMM. Interviewees stated that the meeting did not cause them to directly and knowingly alter their practices the next day, but that their clinical reasoning was affected more indirectly (Table 1, quote 3.2). The reflections helped them to become more mindful about the occurrence and prevention of complications in various types of cases. This was sometimes referred to as ‘clinical expertise’ and was considered to be difficult to observe or measure yet felt to be important and valuable for the entire team (Table 1, quote 3.3). In addition, a weekly discussion of individual cases was felt to be more effective for assessing whether complications occurred (un)expectedly and under what circumstances, to determine what can be learned from this. In contrast, assessing aggregated annual outcomes (e.g., complication or mortality rates) is both useful and mandatory for some purposes, yet was experienced to result in a different type of discussion because it can never be directly related to the specific cases (Table 1, quote 3.4).

**Shared professional values**

By sharing and assessing values regarding clinical, emotional and process-related aspects of delivered care, a form of a professional identity was shaped and enforced in the meeting; thereby contributing to the SMM. To illustrate, the interviewees perceived the meeting as a form of a critical audit of clinical cases, in which the team’s final opinion can differ from formal evaluations based on the more universal standard (Table 1, quote 4.1). As an example, a case could formally be regarded as ‘unsuccessful’, due to many complications or a length of stay longer than the (inter)national benchmark, yet the meeting provided for an opportunity to formulate a more nuanced, collective professional judgement (e.g., discussing how the team responded to complications that arose, or how this complicated course perhaps was still better than anticipated for this individual case). The possible bias posed by doctor-patient relationship in auditing cases was acknowledged but felt to be potentially reduced by other team members questioning and offering a different view on the case (Table 1, quote 4.2). The presence of shared professional beliefs could also be found in the collaboration between, for instance, nurses and surgeons. This could foremostly be seen in the meeting being a moment to reflect on multidisciplinary teamwork as well, providing for a moment to share thoughts on an incident that took place on the ward (Table 1, quote 4.3). The common perception of professional values formed the basis of the shared understanding of fellow team members’ roles, their clinical and organizational responsibilities or communication patterns, and could therefore be considered one of the cornerstones of the SMM.

**Cohesiveness within, and between disciplines**

Interviewees expressed that the meeting increased cohesiveness between nurses and doctors, as well as among the surgical specialists. The opportunity to question fellow experts about their opinion on cases, or about experiences with certain subspecialty-related clinical dilemmas or challenges, was considered supportive for mutual trust and collaboration, besides it being informative as well. This way, cohesiveness within and between disciplines appeared to contribute to the SMM. At the same time, it was also noted that increased cohesiveness was in part a consequence of the SMM, i.e., a consequence of being able to discuss at a certain professional level due to the team having a shared understanding about the team dynamics and functioning. With an SMM effectuating for a team to be ‘on the same page’, this perception could in turn intensify the collaboration and mutual cohesiveness. With that, there seemed to be a reciprocal relation between the SMM and the team’s cohesiveness between team members (Figure 1).

**Proactivity**

Having an SMM seemed to enable the team to develop and support an action-oriented mindset. By discussing similar cases on a frequent basis, interviewees mentioned to recognize opportunities for improvement, as a result of recognizing patterns and becoming aware of recurring issues. Without consciously researching the local data, the reflection on cases, combined with a shared goal of wanting to optimize everyday care, resulted in team members contemplating on altering, for instance, a surgical approach (Table 1, quote 6.1). In the ambition to improve, there was awareness for every complication, irrespective of the severity or preventability (Table 1, quote 6.4). Some interviewees mentioned the difficulty of embedding (large-scale) improvement projects into daily practice, as these require intensive collaboration and cohesion with other caregivers involved. This illustrates how it can be difficult to implement initiatives with others who do not share the same SMM. For instance, it was considered challenging to realize a decrease in postoperative pneumonias or pressure ulcers with clinicians from the Intensive Care Unit, who also provide postoperative care to vascular patients, but have a different understanding of likely postoperative scenarios, and subsequent awareness for risks and opportunities.

**Supplementary Appendixes**

**Appendix S1. The value of the reflective team meeting as an alternative format for M&M**

Different from traditional M&M conferences, this reflective team meeting is based on the principle of discussing everyday care, including all cases. As a result, a large part of the inpatient surgical cases is discussed twice, i.e., both pre- and postoperatively, allowing the team to combine anticipation with evaluation and learning, as is illustrated by the examples in supplementary box 1. Anticipating a scheduled procedure often seems to lead to consulting other specialists or organizing preoperative multidisciplinary team meetings. Moreover, anticipation might pertain the opportunity to prevent certain complications, such as a delirium, yet might naturally also result in the event still occurring, despite all precautions being taken. By discussing every case, the ones that initially might seem ordinary or minimally complicated, so potentially not that interesting to discuss, give rise to reflection as well, such as in example 2.1. In this case, a low severity complication was found to be a stimulus for improvement of practices as well, whereas in M&M, mostly unique or high severity complications are reviewed.(11) Therefore, this reflective team meeting is particularly focused on making routine practices explicit. Explicating how a team manages to create safe patient care on a daily basis, amidst dynamic conditions inherent to the complexity of healthcare, allows for the discussion of more generalist, commonly occurring elements of normal working practices, forming a solid base for the development of shared ideas on how to organize and improve these.(12-14)

Regular reflection on a broad spectrum of outcomes had other effects as well, compared to those described for M&M. A narrow focus on individual performance, as well as the challenge to realize a safe environment with input from all participants, have been observed in M&M,(5, 11, 15-19) whereas this study indicates that the new meeting supported cohesiveness and open interaction among team members. This is likely the result of a safe environment to which sharing a mental model may have helped. Nonetheless, similar to the dilemma that has been addressed in studies on safety culture as well, the question regarding (reflective) team meetings remains as to whether the safe environment is a requirement or a consequence.(20) This study affirms a reciprocal relation, by portraying the mutual relation between the SMM and interpersonal cohesiveness. Even though not formally studied, it is imaginable that more traditional M&M formats could contribute to creating (elements of) an SMM as well, such as shared knowledge about clinical scenarios or surgical procedures. Yet, traditional M&M mostly focus on a selection of complicated cases, which inherently makes it more challenging to have a positive and open discussion, and to ascertain and reinforce everyday practices that have proven to be effective to achieve successful outcomes.

**Supplementary Figures and tables**

**Box S1.** Real case examples of anticipation and evaluation at a surgical reflective team meeting.

| **Examples of anticipation** |
| --- |
| 1.1 *A case anticipated as complex:* An older patient with a fast-growing complex aortic aneurysm was preoperatively anticipated to be at high risk for complications, due to a diminished physical condition and a preexistent cognitive disorder. A preoperative multidisciplinary meeting with anesthesiologists was arranged and the Department of Geriatric Medicine was consulted. The recommendations for the postoperative period included, among other things, fast mobilization with a physiotherapist and taking preventive measures for a delirium. Both the procedure and admission were uncomplicated. The patient was discharged after closely monitoring him longer than required by the national benchmark. During the team meeting, it was debated to what extent this was a case of luck, with the patient not experiencing any complications. However, the team concluded that, as a result of team members being well-informed and attentive towards the possibility of these events to occur, as well as because of all preventive measures taken, this was a case of important teamwork and awareness. |
| **Examples of evaluation and learning** |
| 2.1 *A complex case with a mild complication, but lesson learned*: 4-fenestration endovascular repair (4-FEVAR) was performed to eliminate a complex aortic aneurysm, in a patient that was considered unfit for open repair. The postoperative complication ‘anemia’ was registered, as the patient required a blood transfusion following a periprocedural blood loss of 2 liters. Apart from this complication, the procedure was technically successful, and the patient was discharged after a short stay on the vascular care ward. The complication was classified as low severity and was subsequently discussed in the team meeting. The team contemplated on recent similar cases and concluded that significant blood loss was often the result of major leakage along the sheath of the endovascular device, for which they contacted the manufacturer. Despite the complication being of low severity, it was considered relevant to collectively discuss the case, as this volume of blood loss may be comparable to open repair of such an aneurysm, and there was a need for future improvement of this issue. |
| 2.2 *A complex case without any complications*: A large, complex aortic aneurysm was detected in an older patient, who personally had a strong preference to be treated but was anticipated as at high risk for postoperative complications (e.g., pneumonia or delirium). Moreover, the aneurysm was technically challenging to repair endovascularly. The patient received endovascular treatment, experienced an uncomplicated peri- and postoperative course, and was discharged within two days after the procedure. In reflecting upon this case, the team members considered this an exemplary case of what good quality care could entail. It moreover reinforced that the extensive preparation measures seemed to prove their value, because they had organized multiple preoperative multidisciplinary team meetings to deliberate on this case in advance. This could be taken as a lesson for similar cases in the future. In the previous local M&M format, this case would not have been selected for discussion. |

**File S1.** Topic list for semi-structured interviews with participants (surgeons, residents and nurses) of the reflective team meeting.

Introduction

- Background and objectives of this study
- Information about the interview
- Introduction interviewers and interviewee

Reflective team meeting

- General
  - Regularity of attendance, and last time present at the team meeting
  - Example of a recent clinical case discussed
- Experiences with, and general opinion on the meeting
  - The value for patients / for the team
- Culture in the reflective team meeting
  - Consequences for the team after the introduction of the meeting
  - Collaboration and teamwork
  - Ambiance
  - Liberty to discuss issues
    - E.g., relating to the clinical course, communication, organizational aspects or patient satisfaction
  - Essential topics to discuss in the evaluation of a recently discharged patient
- Learning and improving
  - The ability to learn from the meeting
  - Educational value of discussing:
    - Complications, successful outcomes, logistics, patients’ clinical course
  - Prerequisites for learning
  - Ability to improve as a result of the meeting
- Traditional Morbidity and Mortality conferences
- *Vignette 1: ‘A patient with a pacemaker, requiring an MRI for osteomyelitis’.*
- Influence on everyday work
  - Ways in which the meeting does or does not influence daily work
    - E.g., clinical decision making, arranging discharge, informing a patient, prescribing medication and other
- *Vignette 2: ‘A patient anticipated as complex that experienced a short, uncomplicated stay’.*
- Complications
  - Relation between the meeting and the registration / incidence / prevention of complications
  - Monitoring outcomes
- *Vignette 3: ‘A patient with many complications after a complex procedure’*

**Supplementary References**

1. Tong A, Sainsbury P, Craig J. Consolidated criteria for reporting qualitative research (COREQ): a 32-item checklist for interviews and focus groups. Int J Qual Health Care. 2007;19(6):349-57.

2. de Vos MS, Marang-van de Mheen PJ, Smith AD, Mou D, Whang EE, Hamming JF. Toward Best Practices for Surgical Morbidity and Mortality Conferences: A Mixed Methods Study. J Surg Educ. 2018;75(1):33-42.

3. Kievit J, Krukerink M, Marang-van de Mheen PJ. Surgical adverse outcome reporting as part of routine clinical care. Qual Saf Health Care. 2010;19(6):e20.

4. Verhagen MJ, de Vos MS, Hamming JF. Taking Morbidity and Mortality Conferences to a Next Level: The Resilience Engineering Concept. Ann Surg. 2020;272(5):678-83.

5. de Vos MS, Hamming JF, Marang-van de Mheen PJ. Barriers and facilitators to learn and improve through morbidity and mortality conferences: a qualitative study. BMJ Open. 2017;7(11):e018833.

6. Mohammed S, Ferzandi L, Hamilton K. Metaphor No More: A 15-Year Review of the Team Mental Model Construct. Journal of Management. 2010;36(4):876-910.

7. Mathieu JE, Heffner TS, Goodwin GF, Salas E, Cannon-Bowers JA. The influence of shared mental models on team process and performance. J Appl Psychol. 2000;85(2):273-83.

8. Manges K, Groves PS, Farag A, Peterson R, Harton J, Greysen SR. A mixed methods study examining teamwork shared mental models of interprofessional teams during hospital discharge. BMJ Qual Saf. 2020;29(6):499-508.

9. Cannon-Bowers JA, Salas E. Reflections on shared cognition. Journal of Organizational Behavior. 2001;22:195-202.

10. Weller J, Boyd M, Cumin D. Teams, tribes and patient safety: overcoming barriers to effective teamwork in healthcare. Postgrad Med J. 2014;90(1061):149-54.

11. Xiong X, Johnson T, Jayaraman D, McDonald EG, Martel M, Barkun AN. At the Crossroad with Morbidity and Mortality Conferences: Lessons Learned through a Narrative Systematic Review. Can J Gastroenterol Hepatol. 2016;2016:7679196.

12. McHugh SK, Lawton R, O'Hara JK, Sheard L. Does team reflexivity impact teamwork and communication in interprofessional hospital-based healthcare teams? A systematic review and narrative synthesis. BMJ Qual Saf. 2020;29(8):672-83.

13. Iedema R. Creating safety by strengthening clinicians' capacity for reflexivity. BMJ Qual Saf. 2011;20 Suppl 1:i83-6.

14. Verhagen MJ, de Vos MS, Sujan M, Hamming JF. The problem with making Safety-II work in healthcare. BMJ Qual Saf. 2022;31(5):402-8.

15. Berenholtz SM, Hartsell TL, Pronovost PJ. Learning from defects to enhance morbidity and mortality conferences. Am J Med Qual. 2009;24(3):192-5.

16. Pierluissi E, Fischer MA, Campbell AR, Landefeld CS. Discussion of medical errors in morbidity and mortality conferences. JAMA. 2003;290(21):2838-42.

17. Wachter RM, Shojania KG, Saint S, Markowitz AJ, Smith M. Learning from our mistakes: quality grand rounds, a new case-based series on medical errors and patient safety. Ann Intern Med. 2002;136(11):850-2.

18. Harbison SP, Regehr G. Faculty and resident opinions regarding the role of morbidity and mortality conference. Am J Surg. 1999;177(2):136-9.

19. Bechtold ML, Scott S, Nelson K, Cox KR, Dellsperger KC, Hall LW. Educational quality improvement report: outcomes from a revised morbidity and mortality format that emphasised patient safety. Qual Saf Health Care. 2007;16(6):422-7.

20. Pronovost PJ, Goeschel CA, Marsteller JA, Sexton JB, Pham JC, Berenholtz SM. Framework for patient safety research and improvement. Circulation. 2009;119(2):330-7.
